# Modulation of the Akt Pathway Reveals a Novel Link with PERK/eIF2α, which Is Relevant during Hypoxia

**DOI:** 10.1371/journal.pone.0069668

**Published:** 2013-07-29

**Authors:** Matías Blaustein, Daniela Pérez-Munizaga, Manuel Alejandro Sánchez, Carolina Urrutia, Alicia Grande, Guillermo Risso, Anabella Srebrow, Jennifer Alfaro, Alejandro Colman-Lerner

**Affiliations:** 1 Instituto de Fisiología, Biología Molecular y Neurociencias, Consejo Nacional de Investigaciones Científicas y Técnicas y Departamento de Fisiología, Biología Molecular y Celular, Facultad de Ciencias Exactas y Naturales, Universidad de Buenos Aires, Buenos Aires, Argentina; 2 Fundación Ciencia y Vida, Santiago de Chile, Chile; 3 Facultad de Ciencias Biológicas, Universidad Andrés Bello, Santiago, Chile; Wayne State University, United States of America

## Abstract

The unfolded protein response (UPR) and the Akt signaling pathway share several regulatory functions and have the capacity to determine cell outcome under specific conditions. However, both pathways have largely been studied independently. Here, we asked whether the Akt pathway regulates the UPR. To this end, we used a series of chemical compounds that modulate PI3K/Akt pathway and monitored the activity of the three UPR branches: PERK, IRE1 and ATF6. The antiproliferative and antiviral drug Akt-IV strongly and persistently activated all three branches of the UPR. We present evidence that activation of PERK/eIF2α requires Akt and that PERK is a direct Akt target. Chemical activation of this novel Akt/PERK pathway by Akt-IV leads to cell death, which was largely dependent on the presence of PERK and IRE1. Finally, we show that hypoxia-induced activation of eIF2α requires Akt, providing a physiologically relevant condition for the interaction between Akt and the PERK branch of the UPR. These data suggest the UPR and the Akt pathway signal to one another as a means of controlling cell fate.

## Introduction

Akt (also known as protein kinase B or PKB) is a serine/threonine kinase member of the AGC family of protein kinases, which plays a central role in growth, proliferation, protein translation and cell survival [1]–[3]. Akt is recruited to the plasma membrane by phosphatidylinositol (3,4,5)-triphosphate (PIP_3_) generated by activated PI3K. Once at the membrane, Akt is phosphorylated on Thr308 [4] by PDK1 and on Ser473 by mTORC2 [5]. Akt can also be phosphorylated at other sites, which are important for its kinase activity [6], [7]. Activated Akt phosphorylates multiple targets in the cytoplasm, nucleus, mitochondria and at the surface of the endoplasmic reticulum membrane (ER) [8], [9]. Deregulation of the Akt pathway is associated with a variety of human cancers, and mouse models with activated Akt support its role in cancer development [10]–[12]. Several inhibitors of the Akt pathway have been developed as therapeutic treatments, some of which are currently being tested in clinical trials [13]–[16]. One of these inhibitors is the benzimidazole derivative Akt-IV (also known as ChemBridge 5233705 or Akt inhibitor IV) [17], which has potent anticancer and antiviral activity [18]–[20]. Although the direct target of Akt-IV is not known, it has been proposed to bind the ATP pocket of a kinase upstream of Akt but downstream of PI3K, possibly that of PDK1 [17]. Akt-IV has been shown to inhibit the phosphorylation and activity of Akt. However, at low concentrations, Akt-IV promotes the hyperphosphorylation of Akt [20]. The mechanisms behind these seemingly contradictory effects of Akt-IV on the Akt protein and its antiviral and antiproliferative activities are poorly understood.

Like the Akt pathway, the unfolded protein response (UPR) is involved in the regulation of metabolism, protein translation, cell death and survival [21], [22], and it is thought to be important in the development of different malignant neoplasms such as multiple myeloma, prostate and breast cancer [23], [24]. The accumulation of unfolded proteins in the lumen of the ER triggers a multipronged signal transduction response aimed at reestablishing cellular homeostasis. This includes a rapid reduction in the protein load in the ER, which is accomplished by lowering protein synthesis and translocation into the ER, and an increase in the capacity of the ER to fold proteins by upregulating the expression of foldases and chaperones. If homeostasis cannot be reestablished, the UPR can induce cell death, probably to protect the organism from rogue cells that express misfolded proteins. Three ER stress transducers have been identified: inositol-requiring protein-1 (IRE1), activating transcription factor-6 (ATF6) and protein kinase RNA (PKR)-like ER kinase (PERK). These integral membrane proteins sense the protein folding status in the ER lumen and communicate this information to cytosolic target proteins that translocate to the nucleus to modulate gene expression [22], [25], [26]. The UPR was historically viewed as a stress response system but a growing body of work suggests that it also functions in the maintenance of basal cellular homeostasis [21]. In this view, the UPR could be activated and its output modulated by signals other than misfolded proteins. In accordance with this notion, P58-IPK and a novel cytosolic isoform of BIP have been described to interact and regulate PERK from the cytosolic side [27]–[29].

Both the UPR and the Akt signaling pathways regulate protein translation, albeit in opposing ways. Akt promotes translation by two paths. First, it phosphorylates and activates mTORC1, which in turn inactivates 4EBP by phosphorylating it in at least four sites [2]. Unphosphorylated 4EBP blocks translation by binding to the cap binding protein eIF4E, the rate limiting step in cap-dependent translation. Second, Akt inactivates the glycogen synthetase kinase 3β (GSK3β), the major kinase that phosphorylates and inactivates the eukaryotic initiation factor 2 (eIF2) and its activator, the guanine exchange factor eIF2B [3]. In contrast, the UPR, via PERK, blocks translation initiation by directly phosphorylating one of the subunits of eIF2 trimer, eIF2α. eIF2 containing phosphorylated eIF2α inhibits eIF2B, preventing further activation of eIF2 [26]. Although the UPR and the Akt pathway have long been known to regulate similar cellular systems and influence cell fate, a functional link between the two pathways has only recently emerged. The UPR has been reported to activate [30]–[35] or inhibit [36]–[38] the Akt pathway depending on the nature and severity of the ER insult. It was recently proposed that Akt phosphorylates and inhibits PERK [39], although the role of the Akt pathway in regulating the UPR remains poorly understood.

In this work we used a series of chemical modifiers of the Akt pathway and tested whether Akt signaling can regulate the UPR. We found that Akt-IV strongly activates all the three branches of the UPR, with the PERK branch showing a dependence on the presence and activity of Akt. Our results suggest that Akt is a cytosolic regulator of the UPR and that the signaling between these two pathways may control the balance of pro-survival and pro-apoptotic signals thereby regulating cell fate decisions.

## Results

### Pharmacological modulation of Akt with Akt-IV activates the UPR

**Figure 1:**
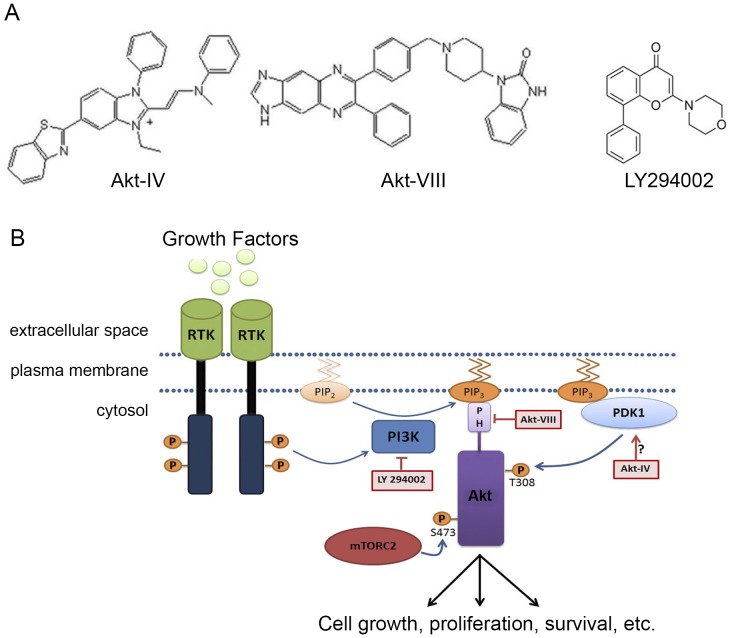
Strategy. (**A**) Chemical structure of the compounds targeting the PI3K/Akt pathway used in this study. (**B**) Scheme of Akt signaling pathway, which regulates cell survival, showing the point of action of the drugs shown in **A**. Akt phosphorylation and activation result from its recruitment to PIP_3_ at plasma membrane, after which it exerts cytoplasmic and nuclear functions. Accumulation of PIP_3_ classically follows ligand (L) binding to tyrosin kinase cell-surface receptors (RTK), adapter proteins (AP) recruitment to RTK and finally, PI3K activation to phosphorylate PIP_2_ to PIP_3_. While Akt can be activated by the UPR it is not known if Akt can also regulate the UPR.

To test the hypothesis that the Akt pathway regulates the UPR, we used a series of small molecules that differentially target the PI3K/Akt pathway (Fig. 1A, B). We used the classic PI3K inhibitor LY294002 [40], Akt-VIII, a direct and selective inhibitor of Akt [41], and Akt-IV. We evaluated the effects of these drugs on the activation of the three branches of the UPR in HEK293T cells in culture.

**Figure 2:**
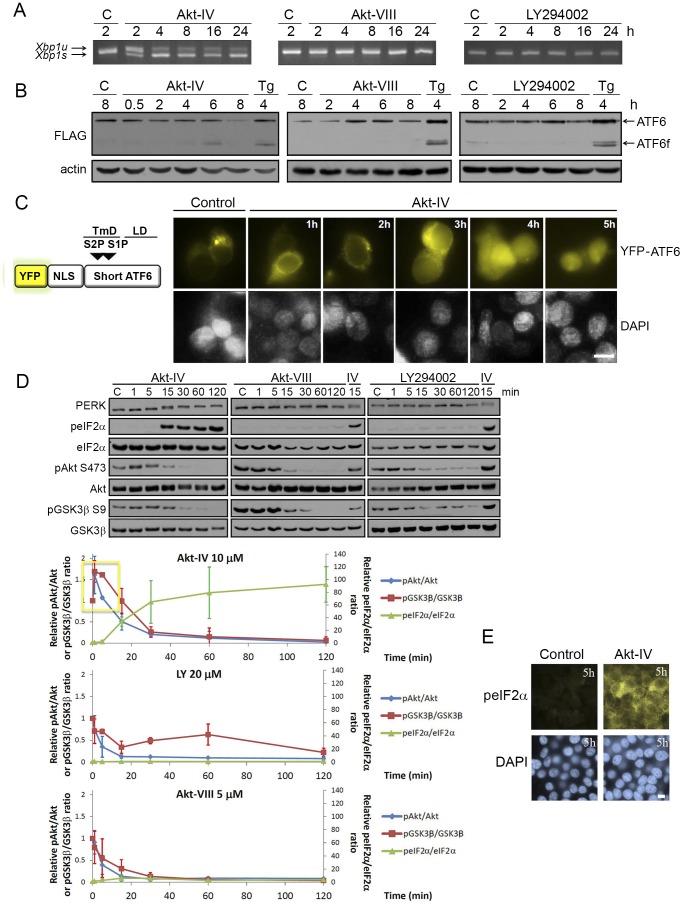
Pharmacological modulation of Akt with Akt-IV activates all three UPR branches: PERK responds first. (**A**) When activated, IRE1 processes *Xbp1* mRNA by a non-conventional cytoplasmic splicing reaction, changing *Xbp1* open reading frame. HEK293T cells were treated with Akt-IV (10 µM), Akt-VIII (5 µM) or LY294002 (20 µM) for the indicated times. *Xbp1* mRNA splicing was detected by RT-PCR. *Xbp1s*: spliced form (activated IRE1); *Xbp1u*: unspliced form (inactive IRE1). (**B**) When activated, ATF6 translocates to the Golgi apparatus where it is cleaved to release a fragment that enters the nucleus where it functions as a transcription factor. HEK293 cells were transfected with ATF6-Flag plasmid and 24 h later they were treated with Akt-IV (10 µM), Akt-VIII (5 µM), LY294002 (20 µM), or thapsigargin (Tg; 100 nM) for the indicated times. Western blots (WB) using antibodies against FLAG and actin are shown for every case (B, upper panel). ATF6: uncleaved protein; ATF6f: cleaved form. (**C**) HEK293T cells were transfected with a plasmid that expresses the YFP-NLS-mATF6short reporter (top, see Fig. S1A for details). Forty-eight hours post-transfection cells were treated for the indicated times with Akt-IV and then fixed, DNA was stained with DAPI and cells were imaged (lower panel); Yellow, YFP-ATF6; Blue, DNA; scale bar, 5 µm. For all cases cells treated with DMSO were used as a control (Control). (**D**) When activated, PERK is autophosphorylated at multiple residues and activated to phosphorylate eIF2α. HEK293T cells were treated with Akt-IV (10 µM), Akt-VIII (5 µM) or LY294002 (20 µM) for the indicated times. Protein extracts were analyzed by WB using the indicated antibodies. Data in the plot corresponds to ratio of phosphorylated total abundance of each of the indicated proteins (normalized to the initial value) in cells treated with the indicated drugs for different times. Error bars correspond to the standard error of three independent experiments. (**E**) HEK293T cells were treated for 5 h with Akt-IV. peIF2α abundance was detected by immunofluorescence. Green, peIF2α; Blue, DNA; scale bar, 5 µm. Data are representative of at least three independent experiments.

Akt-IV triggered a strong and sustained (2 to 24 h) activation of the IRE1 branch, as detected by monitoring the splicing of *Xbp-1* mRNA by RT-PCR (Fig. 2A). Neither LY294002 nor Akt-VIII showed any effects on *Xbp-1* mRNA splicing. We evaluated the activation of the ATF6 branch by measuring the cleavage of a FLAG-tagged ATF6 by western blot (WB) (Fig. 2B) and the re-localization from the ER to the nucleus of a YFP-ATF6 reporter (Fig. S1A). Akt-IV induced both the cleavage of ATF6-FLAG (Fig. 2B) and the re-localization of the fluorescent reporter (Fig. 2C and Fig. S1B). In contrast, neither LY294002 nor Akt-VIII induced the cleavage of ATF6-FLAG (Fig. 2B). Finally, we found that Akt-IV rapidly activated PERK, as assessed by a mobility shift in an SDS-PAGE gel, and the phosphorylation of the PERK target eIF2α (Fig. 2D and 2E). As with the IRE1 and ATF6 branches, LY294002 and Akt-VIII had little or no effect on the PERK branch (Fig. 2D). Even at longer incubation times these compounds failed to activate the PERK branch at levels comparable to those of Akt-IV (Fig. S2). As expected, all three compounds inhibited the phosphorylation of Akt at Ser473 and of its downstream target GSK3β at Ser9 (Fig. 2D), making the effects of Akt-IV on the UPR all the more striking.

We noted that just before PERK branch activation, Akt-IV caused an early transient activation of Akt, as assessed by the increase in phosphorylation of Ser473 of Akt and on Ser9 of GSK3β (Fig. 2D). This activation peaked between 1 and 5 minutes post stimulation with Akt-IV and was specific to this compound since neither LY294002 nor Akt-VIII provoked such behavior (Fig. 2D). If this transient activation of Akt is important for the induction of eIF2α phosphorylation needs further confirmation.

### eIF2α phosphorylation induced in response to Akt-IV depends on PERK and Akt but not on PI3K

**Figure 3:**
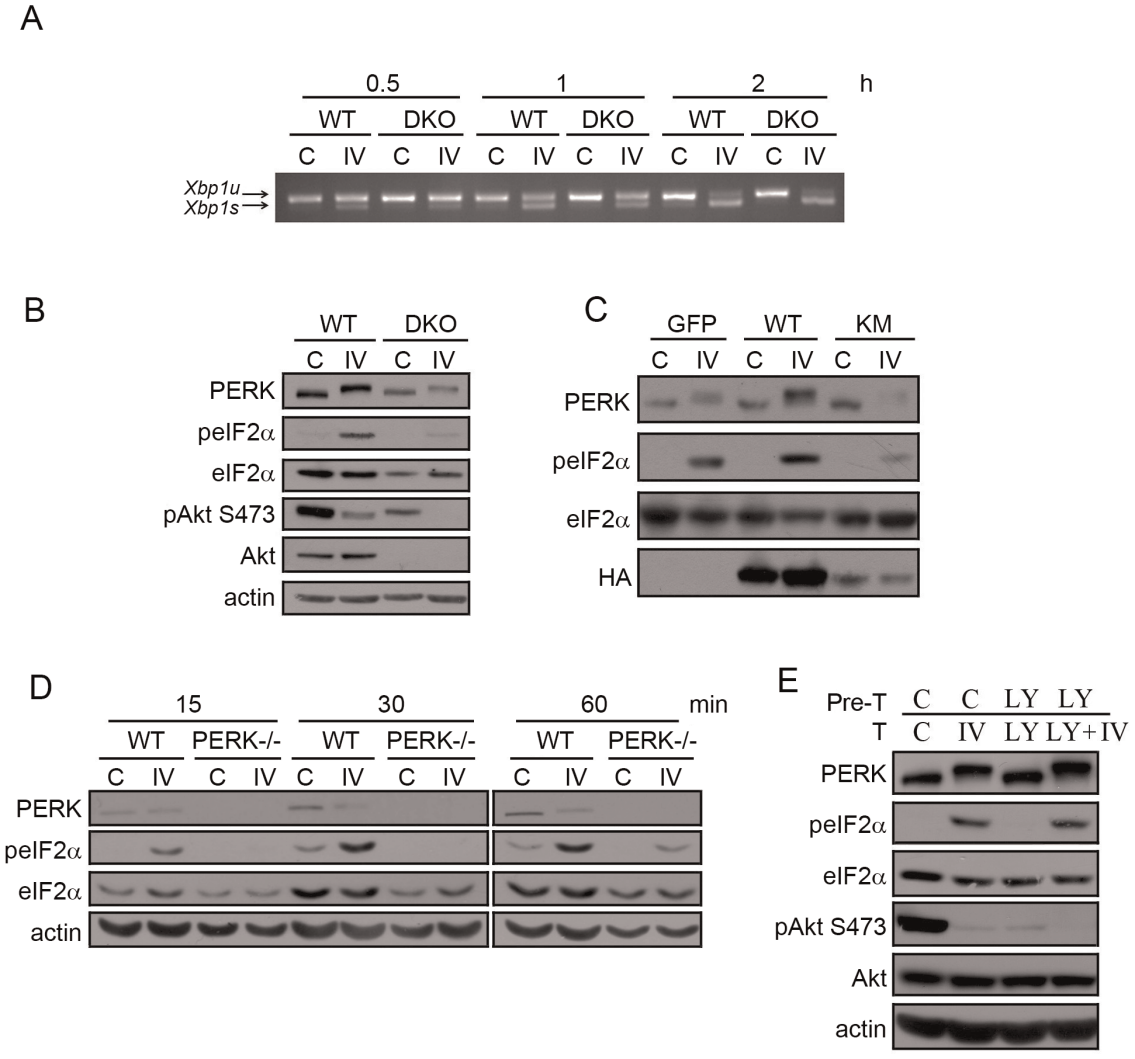
Akt-IV stimulation of eIF2α phosphorylation is Akt- and PERK- dependent but PI3K-independent. (**A**) MEF WT or Akt DKO cells were treated with Akt-IV (IV; 10 µM) for the indicated times. *Xbp1* mRNA splicing was detected by RT-PCR. *Xbp1s*: spliced form (activated IRE1); *Xbp1u*: unspliced form (inactive IRE1). (**B**) MEF WT or Akt DKO cells were treated with Akt-IV (IV; 10 µM) for 1 h. Protein extracts were analyzed by WB using the indicated antibodies. The fold change in peIF2α/eIF2α ratio induced by Akt-IV was quantified for three independent experiments. On average, this fold change was reduced to 15% of the original effect in MEF Akt DKO cells compared to WT cells (11.0 vs 2.6). (**C**) MEF WT cells were transfected with HA-Akt or HA-Akt KM plasmids. Forty-eight hours post-transfection cells were treated with DMSO (C) or with Akt-IV (IV; 10 µM) for 1 h. A GFP expressing plasmid was used as a transfection control. Protein extracts were analyzed by WB using the indicated antibodies. (D) MEFs WT or PERK−/− were treated with Akt-IV (IV; 10 µM) for the indicated times. Protein extracts were analyzed by WB using the indicated antibodies. (**E**) HEK293T cells were pretreated with DMSO (C) or LY294002 (LY; 20 µM) for 30 min and then treated for 1 h with DMSO, or Akt-IV (without removing the corresponding pre-treatment). Protein extracts were analyzed by WB using the indicated antibodies. Data are representative of at least three independent experiments.

Since all three compounds work as Akt inhibitors (Fig. 2D), but only Akt-IV activates the UPR, we tested the possibility that Akt-IV regulates the UPR in an Akt-independent manner. To do that, we evaluated the effects of Akt-IV on mouse embryonic fibroblasts (MEFs) lacking Akt1 and Akt2, two of the three isoforms of Akt (Akt DKO, Fig. S3). These cells have no basal activation of *Xbp-1* mRNA splicing nor have they increased basal PERK activity as judged by eIF2α phosphorylation. Treatment of Akt DKO cells with Akt-IV induced the splicing of *Xbp-1* as well as the mobility shift of PERK to a similar extent as in wild type MEFs (Fig. 3A and 3B) indicating that these effects are independent of Akt1 and Akt2. However, the absence of these two isoforms impaired the capacity of Akt-IV to induce the phosphorylation of eIF2α (Fig. 3B) showing that in the case of the effect on eIF2α, Akt1 or Akt2 kinases were required. The remaining Akt and pAkt signal observed by western blot and immunofluorescence in these cells (Fig. 3B and Fig. S3) is likely due to the fact that Akt DKO cells still express Akt3. To test whether the kinase activity of Akt is required as well, we transfected wild type MEFs with a vector containing a dominant negative kinase dead allele of Akt (HA-Akt-KM) [42] and determined the effect of Akt-IV on eIF2α phosphorylation. As in Akt DKO cells, the phosphorylation of eIF2α induced by Akt-IV was greatly reduced in cells overexpressing HA-Akt-KM compared to wild type cells (3 fold decrease in peIF2α/eIF2α levels compared to control, Fig. 3C) whereas the mobility shift of PERK was unaffected. Moreover, over-expression of wild type HA-Akt potentiated the effect of Akt-IV on eIF2α phosphorylation (3 fold increase in peIF2α/eIF2α levels compared to control, Fig. 3C), without affecting phosphorylation of eIF2α in untreated cells.

Thus far, we have shown that Akt is necessary for eIF2α phosphorylation, but not for the mobility shift of PERK. This raises the possibility that Akt might cause this effect in a PERK independent manner. In fact, eIF2α is known to be phosphorylated independently of the UPR by GCN2, HRI and PKR [43]. To test whether PERK was the kinase responsible for phosphorylating eIF2α in the presence of Akt-IV, we treated PERK knock out MEFs (PERK−/−) with Akt-IV (Fig. 3D). We found that the ability of Akt-IV to induce eIF2α phosphorylation was greatly impaired in PERK−/− MEFs as compared to wild type cells. The residual phosphorylation of eIF2α visible at later time points (1 h) is likely due to one or more of the other eIF2α kinases [44].

Our data are consistent with a model in which Akt-IV promotes activation of the PERK/eIF2α branch by triggering Akt kinase activity. To test whether the effects of Akt-IV on PERK and eIF2α require the canonical activation of Akt by PI3K, we pre-treated wild type MEFs with the PI3K inhibitor LY294002 before stimulating with Akt-IV. As expected, this pre-treatment inhibited the phosphorylation of Akt on Ser473 (Fig. 3E). However, it did not affect the ability of Akt-IV to induce the phosphorylation of eIF2α (Fig. 3E).

Taken together, these results demonstrate that the effect of Akt-IV on eIF2α phosphorylation is PI3K-independent but Akt- and PERK-dependent.

### PERK is an Akt substrate

**Figure 4:**
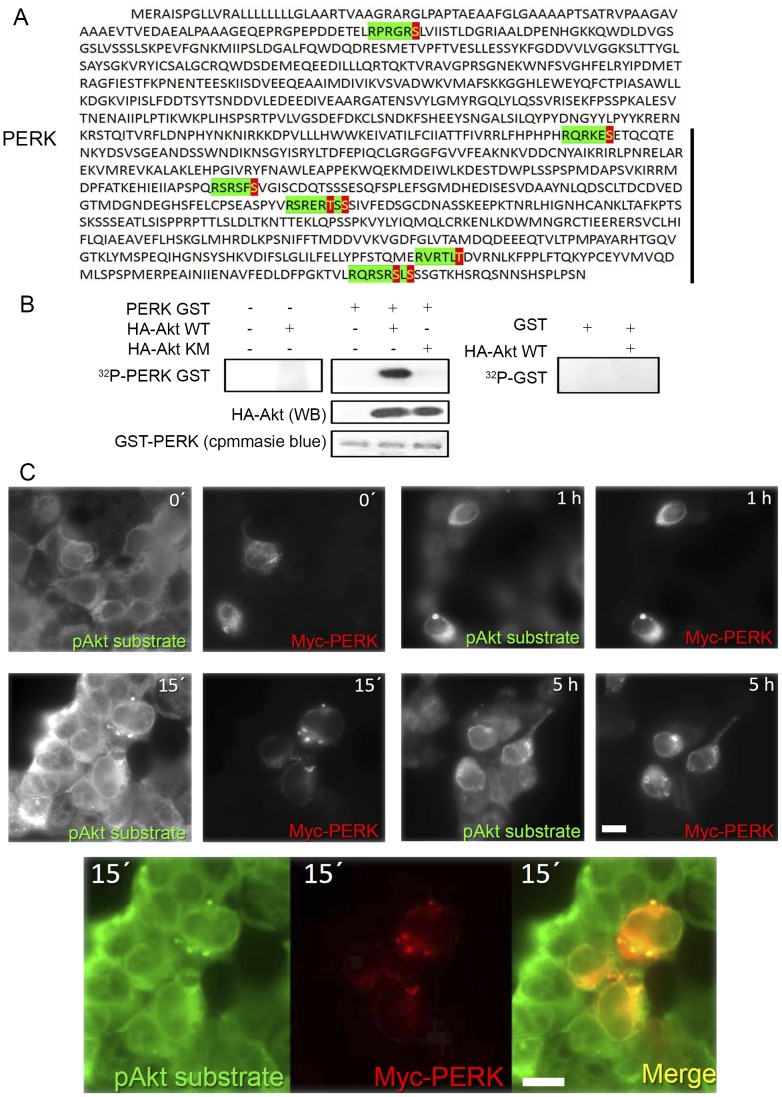
Akt is a PERK kinase. (**A**) Human PERK gene containing eight sequences that match the Akt consensus (RxRxxS/T) seven of which are located in its cytosolic domain (black bar). Green: Akt consensus sequence. Red/Yellow: Akt phosphorylation site. (**B**) WT and KM HA-Akt mutant protein was incubated with 10 µCi of [γ-^32^P] ATP for 30 min and with 1 µg of GST or PERK GST protein as substrate. Phosphate incorporation was analyzed by SDS-PAGE and autoradiography. Akt levels were determined by WB while GST-PERK levels were revealed by Coommassie blue staining. (**C**) HEK293T cells were transfected with pCDNA-Myc-PERK and treated for 15 min with Akt-IV (10 µM). Cells were fixed and immunostained for pAkt substrate and Myc tag. Green, pAkt substrate; Red, Myc-PERK; scale bar, 5 µm. Data are representative of at least three independent experiments.

One possibility is that the eIF2α phosphorylation elicited by Akt-IV is due to the activation and phosphorylation of PERK by Akt or one of its substrates. Alternatively, Akt and PERK could act in parallel, such that the phosphorylation of eIF2α in the presence of Akt-IV requires both. If the former model were true, PERK might be a direct target of Akt, what could be detected in an *in vitro* kinase assay. In support of this notion, sequence analysis revealed that while neither IRE1 nor ATF6 contain putative cytosolic Akt phosphorylation sites, human PERK contains eight sequences that match the Akt consensus sequence (RxRxxS/T) [45]. Seven of these sequences, conserved in rats and mice, are located in its cytosolic domain (Fig. 4A) and thus are accessible to Akt. Interestingly, three of these sites (human S554, S1093 and S1095) have been found to be phosphorylated *in vivo* according to PhosphoSitePlus® [45], [46]. An *in vitro* kinase assay with the wild type HA tagged Akt1 kinase domain immunoprecipitated from transfected HEK293T cells and a recombinant catalytically inactive fragment of PERK (aa 537 to 1114) revealed that, indeed, Akt can phosphorylate PERK (Fig. 4B). Immunoprecipitated kinase dead HA-tagged Akt1 kinase domain failed to phosphorylate PERK, confirming the dependence on Akt activity (Fig. 4B).

Supporting an in vivo role of Akt in phosphorylating PERK, fluorescence microscopy experiments with Akt1-CFP and the ER membrane marker ATF6-YFP confirmed previous reports that, in addition to localizing to the cytosol, nucleus and plasma membrane, Akt localizes to the ER membrane (Fig. S4). Furthermore, we detected co-localization of PERK and phosphorylated Akt substrates in cells transfected with myc-PERK and immunostained with an anti-myc antibody and an antibody that recognizes proteins containing a phosphorylated Akt target motif (RxRxxS*/T*) [47] (Fig. 4C). In untreated cells, we detected a structured reticulated cytoplasmic staining with the phospho-Akt substrate antibody. The rather strong signal is likely due to high Akt activity normally present in HEK293T cells [48]. At the same time, the PERK-myc signal was detected in only a few cells due to the low transfection efficiency of this construct. The time-course of the signal revealed by the phospho-Akt substrate antibody showed a peak and decline behavior: after an initial increase seen 15 minutes post treatment, the signal diminished to levels below those seen in untreated cells. This behavior is similar to what we observed by western blot using anti phospho-Ser473-Akt (Fig. 2D), supporting the idea that this antibody was a good reagent to follow in vivo Akt activity. In contrast to the majority of cells, those cells that overexpressed PERK-myc did not exhibit this peak and decline behavior. In these cells, the phospho-Akt substrate signal concentrated in the same spots where the myc-signal was detected. These results indicate that PERK might be a direct target of Akt in vivo.

### Akt-IV induces apoptosis in a UPR-dependent manner

**Figure 5:**
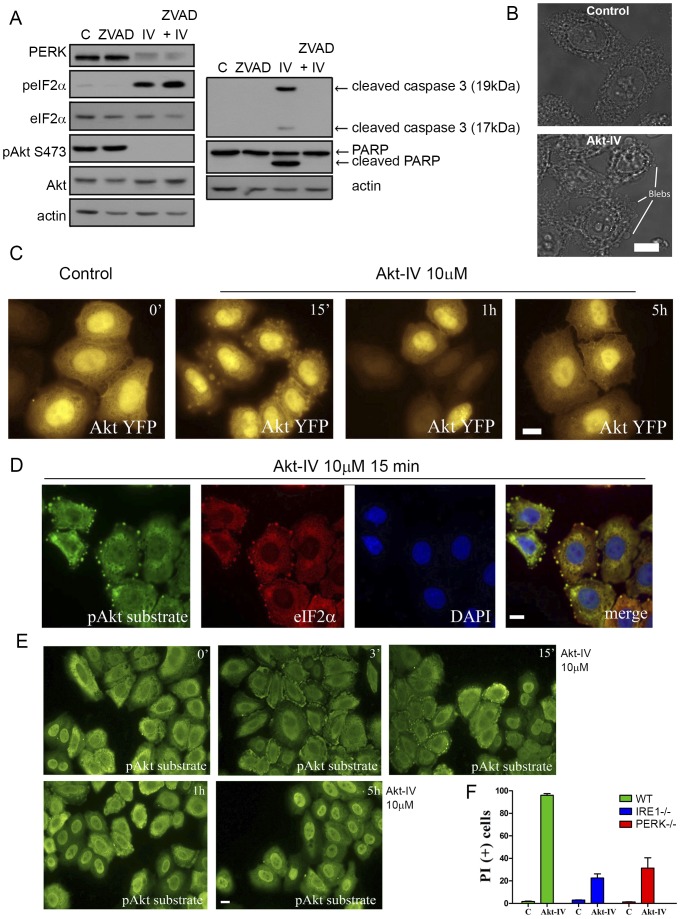
Akt-IV induces cell blebbing and a UPR dependent cell death. (**A**) HEK293T cells were treated with DMSO (C), 100 µM of the caspase inhibitor ZVAD, 10 µM Akt-IV (IV) or both. PERK mobility, eIF2α phosphorylation, Akt phosphorylation on Ser473, caspase 3 cleavage and PARP cleavage were detected by WB. (**B**) Transmission images of HeLa cells treated for 15 min with DMSO (15 min) or with Akt-IV (10 µM), with blebs indicated. Bleb formation was clearly observed in HeLa and MEF cells but could not be detected in HEK293T cells. (**C**) YFP channel images of HeLa cells transfected with pAkt1-YFP plasmid and then treated for the indicated times with Akt-IV (10 µM). Akt1-YFP can be detected in blebs after 15 min of treatment. (**D**) HeLa cells were treated for 15 min with Akt-IV. Cells were fixed and immunostained against pAkt substrate/Alexa Fluor® 488 and total eIF2α/Alexa Fluor® 594. Green, pAkt substrate; Red, eIF2α; Blue, DNA. scale bar, 5 µm. (**E**) HeLa cells were treated for different times with Akt-IV (10 µM) and then cells were fixed and immunostained for pAkt substrate/Alexa Fluor® 488; scale bar, 5 µm. (**F**) MEF WT, IRE1^−/−^ or PERK^−/−^ were treated with Akt-IV (10 µM) for 12 h. Cell viability was measured by flow cytometry using propidium iodide. Data are representative of at least three independent experiments.

Both the Akt pathway and the UPR control cell fate by inducing pro-survival and/or pro-apoptotic signals. Akt-IV has also been described to induce cell death in different cell types [49]–[52]. To determine the effects of Akt-IV on apoptotic cell death in our system, we treated HEK293T cells for 7 hours with Akt-IV and determined by WB the extent of caspase 3 and PARP cleavage. Akt-IV caused strong activation (cleavage) of caspase 3 and PARP, which were blocked by the caspase inhibitor ZVAD (Fig. 5A). Consistent with these results, we found that Akt-IV treatment induces the formation of cell blebs (Fig. 5B). These transient globular protrusions of the plasma membrane are seen at the onset of the certain stress-associated processes, such as cell injury, cell invasion, hypoxia, high concentrations of free radicals and apoptosis in some cell lines [53]. Interestingly, Akt-YFP transiently localizes to these blebs in HeLa cells within 15 minutes of treatment with Akt-IV (Fig. 5C). Consistently, phosphorylated Akt substrates and eIF2α also exhibit a transient enrichment in cell blebs after treatment with Akt-IV treatment (Fig. 5D and 5E), but not with other inhibitors such as Akt-VIII (Fig. S5).

To determine the role of the UPR in the cell death induced by Akt-IV, we evaluated the viability of wild type, IRE1 (IRE1−/−) and PERK (PERK−/−) knock out MEFs using a propidium iodide incorporation assay measured by flow cytometry. Cell death induced by a 12 h treatment with Akt-IV was greatly reduced in the absence of IRE1 or PERK (Fig. 5F), indicating that one of the mechanisms by which Akt-IV induces cell death is UPR dependent.

### Akt mediates PERK/eIF2α activation induced by hypoxia

The internal environment of solid tumors can be hypoxic and can lead to the activation of mechanisms within the tumor to cope with this stress. In general, adaptation to hypoxia is associated to poor response to therapy [54]. During hypoxia, Akt is activated by phosphorylation of Akt in the non-canonical Thr450 by JNK kinase [55], [56]. In the same conditions, eIF2α is phosphorylated in a PERK-dependent manner [57]. Furthermore, hypoxia is associated with cell blebbing and cell death [58]. Thus, given our results, we hypothesized that Akt might be required for eIF2α phosphorylation during hypoxia.

**Figure 6:**
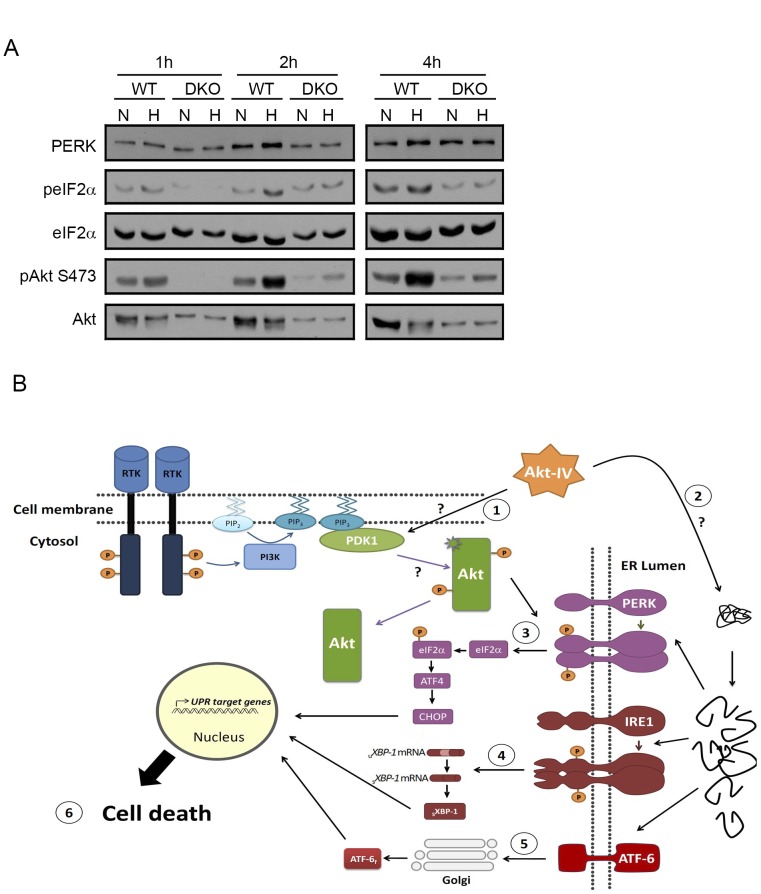
A physiological link between Akt and PERK/eIF2α. (A) WT or Akt DKO MEF cells were subjected to normoxia (C) or hypoxia (0.1%±0.1 O_2_) (H) for the indicated times. Protein extracts were analyzed by WB using the indicated antibodies. The fold change in peIF2α/eIF2α ratio induced by hypoxia was quantified for two independent experiments. On average, this fold change was reduced to 0, 40 or 60% of the original effect in MEF Akt DKO cells compared to WT cells (1 h, 2 h and 4 h, respectively). (B) A model summarizing our results. Akt-IV (or other stimuli, such as hypoxia) targets an unknown kinase, possibly PDK1, triggering apoptotic cell blebbing and activating Akt in a PI3K-independent manner (1). Subsequently, UPR is activated (2). Akt presence and activity are necessary for eIF2α phosphorylation due to the existence of a connection between Akt and PERK/eIF2α signaling pathways. IRE1 (4) and ATF6 (5) are activated are later times. At the end, activation of IRE1 and PERK and dephosphorylation of Akt and GSK3β are associated with cell death (6).

To test this hypothesis, we incubated wild type and Akt DKO MEF cells in low oxygen conditions (0.1% O_2_) for 1, 2 or 4 hours and evaluated the levels of eIF2α phosphorylation. In wild type MEFs, hypoxia induced an increase in phosphorylated eIF2α, but not a PERK mobility shift. It is possible that PERK autophosphorylation under these conditions is either transient or low. However, in Akt DKO MEFs, hypoxic conditions did not affect neither PERK nor eIF2α phosphorylation (Fig. 6A). This result supports our idea that Akt mediates PERK/eIF2α activation during hypoxia.

## Discussion

Here we report the finding of a link that connects Akt with the PERK branch of the UPR. Our results also shed light into the molecular mechanism of action of Akt-IV, a drug with anticancer and antiviral properties with therapeutic potential. We provide evidence supporting the idea that a non-traditional activation of Akt leads to PERK-dependent phosphorylation of eIF2. And more important, we also show that this connection is relevant during a physiological condition such as hypoxia.

Recently, Mounir *et al.* provided initial evidence that Akt regulates PERK signaling [39]. Contrary to our results, the authors reported that Akt phosphorylates and inhibits PERK function. They observed higher levels of eIF2α phosphorylation in Akt DKO MEF cells compared to wild type cells, consistent with Akt being a PERK inhibitor. We have not observed these differences (Fig. 3B), although our source of knock out cells was different [59], [60]. In contrast, we found that the overexpression of a dominant negative form of Akt in wild type MEFs largely blocked eIF2α phosphorylation in response to Akt-IV, consistent with a stimulatory, rather than an inhibitory, action of Akt kinase over PERK (Fig. 3C). Additionally, the authors described that treatment with other Akt inhibitors like Akt-VIII and LY294002 also induced phosphorylation of eIF2α after several hours of treatment. We only detected an effect using the Akt-IV compound (occurring in minutes), with marginal effects using the other Akt inhibitors. It is worth mentioning that they used a very high concentration of Akt-VIII (ten times higher than us and more than twenty times higher than in the paper that originally described Akt-VIII [41]). One potential explanation for our discrepancies is that Akt might phosphorylate different residues of PERK depending on the cell context; some of these could be stimulatory while others inhibitory. This idea would be consistent with the presence of several consensus sites for Akt phosphorylation in the cytosolic domain of PERK (Fig. 4A), and some of which have been shown to be phosphorylated *in vivo* [46], [61]. It has been shown that in some cell contexts a prolonged treatment with PI3K inhibitors reactivates Akt to some extent [62]. Thus, the delayed effect of Akt-VIII and LY294002 on eIF2α reported by Mounir et al. may be due to a nontraditional reactivation of Akt instead of inhibition. Further work will be needed in order to address this issue. Nonetheless, it seems now confirmed that Akt targets and phosphorylates PERK modulating its activity and establishing a connection between these two pathways.

We found that in response to Akt-IV, cells develop cell blebs, to which Akt relocalizes in a transient manner, at a time coincident with the onset of eIF2α phosphorylation (Fig. 2C and 5B–C). Determining if the formation of these blebs is important for this action of Akt will require future work. Nevertheless, this finding supports the current view that Akt-IV probably targets PDK1 [17], since it has been shown that PDK1 is involved in cell blebbing [63]. In the same line, though it needs further confirmation, it seems possible that the activation of the UPR by Akt-IV is via PDK1. In support of this hypothesis, other pharmacological modulators of PDK1 also activate the UPR pathway [64]–[66]. Since these compounds, like Akt-IV, activate Akt1 independent branches (such as IRE1), it seems likely that PDK1 targets the UPR via Akt dependent and independent pathways.

Several non-canonical mechanisms of Akt activation have been recently reported, such as Akt ubiquitination and Akt phosphorylation in residues different from Thr308 and Ser473. In fact, many kinases, including JNK, Ack1, ERK, CaM kinase and PKA are able to phosphorylate Akt, and some of them do so independently from PI3K activity [7], [52], [67]–[71]. Remarkably, in the case of non-traditional activation of Akt induced by Akt-IV, because of the induced eIF2α phosphorylation, it would promote translation inhibition, contrary to the classic function of Akt in stimulating protein synthesis. Rapid and transient activation of the classic Akt pathway (phosphorylation in S473 and of Akt major target GSK3β) precedes persistent activation of PERK/eIF2α pathway, which lasts even after inhibition of Akt (Fig. 2D). This can be explained both by a persistent non-canonical activation of Akt (independent of S473 phosphorylation) and/or by a transient non-canonical activation of Akt, which phosphorylates alternative targets like PERK, which in turn displays a low dephosphorylation rate.

We speculate that both cell death and translation inhibition induced by Akt-IV could be associated with either the late Akt dephosphorylation at S473, as has been described by the group of Luo *et al* [72] or with a non-canonical activation Akt, directing its kinase activity to alternative targets, such as PERK. We show that the absence of PERK greatly reduces cell death induced by Akt-IV, further supporting this alternative (Fig. 5F). Independently of whether IRE1−/− and PERK−/− cells have a reduced apoptosis potential or not, what it is clear is that Akt-IV needs the presence of these proteins to induce cell death. Here, we also show that Akt-IV displays effects that are independent of Akt, such as IRE1 dependent Xbp1 mRNA splicing and PERK electrophoretic mobility shift. We speculate that Akt-IV –perhaps mediated by PDK1 as suggested above- might in fact cause protein unfolding at the ER lumen in an Akt independent manner, affecting all the branches in different manners. In this regard, the shift induced by Akt-IV on PERK mobility in an SDS-PAGE persisted on Akt DK0 cells as well as in cells transfected with HA-Akt-KM (Fig. 3B and 3C), indicating that the phosphorylation of PERK induced by Akt-IV responsible for the shift is independent of the presence of Akt1/2. In addition, this result also showed that the classic mobility shift might not always indicate full PERK activation, since in this case, even though the PERK band shifted, phosphorylation of eIF2α was greatly reduced. Thus, we speculate that Akt signaling activates PERK in a manner that might complement the activation associated with the classical mobility shift.

The design of drugs that target different components of the Akt signaling pathway is of great value for the therapeutic treatment of cancer [13]–[17], [50], [73], [74], and Akt-IV has been shown to exhibit potent anticancer and antiviral activity [13]–[17], [50], [73], [74]. For this reason, new analogues of Akt-IV have been designed with enhanced antiviral/antiproliferative activity and low cytotoxicity in normal cells [50]. Dunn *et al.* found that Akt-IV elicited Akt phosphorylation and blocked viral replication. They proposed that Akt-IV antiviral activity was unrelated to its action on Akt [17], [20]. They based this conclusion solely in their observation that other drugs, like LY294002, neither have this antiviral action nor prevented it, despite blocking Akt phosphorylation, similar to what we observed for Akt-IV and the UPR activation (Fig. 2A–D). However, using MEF Akt DKO cells and a dominant negative form of Akt (Fig. 3A and B), we found that activation of the PERK branch actually required Akt kinase activity. In this regard, it is worth mentioning that the UPR and PERK in particular, play an important role in the expression of viral proteins [75]–[77]. Thus, in the light of our results it would be interesting to test if the antiviral activity of Akt-IV does indeed depend on Akt especially on this newly discovered Akt/PERK pathway.

In summary, our data suggests that Akt-IV triggers cell blebbing and a rapid and non-traditional activation of Akt, which stimulates the PERK branch of the UPR. Akt-IV also activates the IRE1 and ATF6 branches, maybe through the promotion of unfolded proteins at the ER. Akt-IV also promotes a late inhibition of Akt traditional activity (evaluated by loss of pAkt S473 and pGSK3β S9 phosphorylation) which in combination with the UPR eventually result in cell death via apoptosis (Fig. 6B). It has been previously shown that PERK-mediated phosphorylation of eIF2α during hypoxia is important for long-term survival [57]. Here we found that Akt is necessary for eIF2α phosphorylation in these conditions (Fig. 6A), providing an important physiological context for the Akt-PERK interaction. The link between Akt and UPR pathways suggests a constant feedback between them, since ER stress and UPR have already been shown to alter Akt activity [24], [35], [78], [79]. This feedback provides a mechanistic basis for previous suggestions that UPR plays a key role in the maintenance of basal cellular homeostasis to a level far beyond to its role in ER protein folding [21], [24]. Cross-talk between these key signaling cascades is not surprising since both pathways play crucial functions in apoptosis, cell survival, protein translation and tumor growth [10]–[12], [23], [24]. Consistently, the connection between ER stress and the Akt downstream target mTOR was recently highlighted [80]. We suggest that the Akt/UPR connection functions as a master control mechanism of cell decision-making, illustrating the remarkable flexibility of signaling pathways.

## Materials and Methods

### Cell Culture

HEK293T, HEK293, MEF, HeLa, MCF7 and COS7 cells were grown in high glucose (4.5 g/L glucose) Dulbecco's modified Eagle's medium (DMEM, Invitrogen) supplemented with 10% fetal bovine serum (FBS) and penicillin/streptomycin (100 units/ml and 100 µg/ml respectively, Invitrogen) in all cases. HEK293T and MEF cells also have 110 mg/L of sodium pyruvate. MEF WT and Akt DKO were obtained from Yang Luo, *et al* [59]. MEF WT, IRE1^−/−^ and PERK^−/−^ [81] were provided by Walter, P. (UCSF, USA). MEF GCN2^−/−^ [82] were provided by Koromilas, A (Mc Hill University, Canada).

### Chemicals, reagents and treatments

Akt-IV (10 µM), Akt-VIII (5 µM) and LY294002 (20 µM) were obtained from Calbiochem. Thapsigargin (Tg) was obtained from Sigma. Caspase Inhibitor Z-VAD-FMK (ZVAD) (100 µM) is from Promega. The cells were plated (2×10^5^ cells per well for 6 well plates, 4×10^4^ cells per well for 24 well plates and 1×10^4^ cells per well for 96 well glass-bottom imaging plates) and grown for 24 h before transfection and/or treatment for the specified times.

### Plasmids and Transfections

ATF6-FLAG reporter [83], pCMV6-HA-Akt and pCMV6-HA-Akt KM [84] has been previously described. pGEX4T-1-PERK K618R, used for bacterial expression, pCDNA-Myc-PERK and pBABE-Myc-PERK used for eukaryotic cell expression were obtained from Addgene. All cell lines were transfected with Effectene (Qiagen) or Lipofectamine (Invitrogen) according the manufacture instructions.

### Construction of fluorescent reporters

YFP-NLS-mATF6short reporter was constructed by PCR amplification of mouse ATF6 transmembrane and ER luminal domains (short ATF6). Forward primer contained an SV40 NLS sequence upstream of ATF6 specific sequence. This PCR product was digested with *Hind*III and cloned into pEYFP-C1 (Clontech). The primers used were as follows:

mATF6 For:

5′ AATAAAAGCTT TCGCCACC ATGCCAAAAAAGAAGCGTAAGGTCGACGAGGTGGTGTCAGAG 3′

mATF6 Rev:

5′ AATAAAAGCTTCTGCAACGACTCAGGGAT 3′

Akt1-CFP fusion proteins were constructed by PCR amplification of mouse Akt1 followed by digestion with *Hind*III and cloning into pECFP-N1 (Clontech). The primers used were as follows:

mAkt1-FP For:

5′ AATAAAAGCTT TCGCCACCATGAACGACGTAGCCATT G 3′

mAkt1-FP Rev:

5′ AATAAAAGCTTGGCTGTGCCACTGGCTGAG 3′

### RNA Isolation and RT-PCR Amplifications

RNA purification from cultured cells was carried using the RNAeasy kit, (Qiagen, Germany). PolyA mRNA was reverse-transcribed using M-MLV reverse transcriptase (Invitrogen, Carlsbad, CA). cDNA was used as template for PCR amplification across the fragment of the Xbp-1 cDNA bearing the intron target of IRE1α ribonuclease activity. Primers used included: murine Xbp-1 sense 5′-GAACCAGGAGTTAAGAACACG-3′ and antisense 5′-AGGCAACAGTGTCAGAGTCC-3′; human Xbp-1, sense 5′-TTACGAGAGAAAACTCATGGC-3′ and antisense 5′-TCCAAGTTGTCCAGAATGC-3′. PCR conditions were: 95°C for 5 min; 95°C for 30 sec; 56°C for 30 sec; 72°C for 30 sec; 72°C for 5 min with 30 cycles of amplification. PCR products were resolved on a 3% agarose/1× TAE gel.

### Western Blot Analysis

Protein extract preparation and western blot analysis were performed as previously described [85]. Primary antibodies used against PERK, peIF2α S51, eIF2α, pAkt S473, Akt, pGSK3β S9, HA, pAkt substrate, PARP and caspase 3 (all from Cell Signaling Technology), GSK3β (clone H76, Santa Cruz), FLAG and actin (1/10000) (Sigma), all were used at 1/1000 at least that something else were indicated. To quantify the bands obtained via Western blot analysis, we applied ImageJ software based analysis (http://imagej.nih.gov/ij/).

### Cell Imaging and Immunofluorescence

For fluorescent reporter assays and immunofluorescence, cells were plated into 96 well glass-bottom imaging plates and transfected -when indicated- 24 h later with lipofectamine according the manufacter instructions. After treatment, cells were fixed as described [86] with 4% paraformaldehyde in PBS for 5 min at room temperature, washed twice with PBS, permeabilized for 5 min with 0.2% Triton X-100 in PBS and blocked for 30 min with 3% BSA in PBS. After incubation for 1 h with primary and secondary antibodies (Alexa 594 or Alexa 488 labeled anti-mouse antibodies) in blocking solution for 1 h each, cells were extensively washed with PBS and nuclei were stained with DAPI. Images were captured on an Olympus IX-81 fluorescence microscope equipped with a 60× oil immersion objective and a Coolsnap HQ^2^ CCD camera (Photometrics). Acquisition was carried out with MetaMorph® Microscopy Automation & Image Analysis Software.

### Expression of recombinant proteins, immunoprecipitation and PERK *in vitro* Phosphorylation Assays

Expression and purification of GST and GST fusion proteins as well as Akt in vitro kinase assays were performed as described [86]. Briefly, GST and GST-PERK (K618R) were over-expressed in the *E. coli* Rosetta pLysS BL21 strain (Novagen) by incubation at 37°C until the optical density was 0.5 and induction with isopropyl-β-thiogalactopyranoside during 3 h. Following bacterial lysis, soluble proteins were affinity purified under nondenaturing conditions, on a glutathione-sepharose 4B-CL resin (Amersham-Pharmacia) according to the manufacturer instructions. For Akt kinase assays, HEK293T in 100-mm plates were transfected with pCMV6-HA-Akt, pCMV6-HA-Akt KM or were mock transfected. After 48 h cells were washed with cold PBS and lysed at 4°C in Akt lysis buffer (20 mM Tris-HCl pH 7.5, 137 mM NaCl, 1% Triton X100, 10% glycerol, 20 mM NaF, 2 mM sodium vanadate, 1 mM DTT and 25 mM β-glycerophosphate plus a protease inhibitor cocktail (Roche)). WT and KM mutant HA-Akt were immunopurified from cleared lysates with an anti-HA antibody (MMS-101R, Covance) and Gamma-Bind Sepharose beads (Santa Cruz Biotechnology). Lysates from mock transfected cells were subject to the same procedure and used as a negative control. After extensive wash, the immunoprecipitate was incubated with 10 µCi of [γ-^32^P] ATP for 30 minutes in 30 µl kinase buffer (20 mM HEPES pH 7.4, 10 mM MgCl2, 10 mM MnCl2, 1 mM DTT, 20 µM ATP) at 25°C using 1 µg of GST or GST fusion protein as substrate. The reactions were terminated by addition of 5× sample buffer and boiling. Phosphate incorporation was analyzed by sample electrophoresis on a 12% SDS-PAGE and autoradiography.

### Cell viability assay

After the corresponding treatment the cells were incubated with 1 mg/ml of propidium iodide (PI). PI incorporation was measured after 15 min by flow citometry using FACScanto II cytometer and FACS DIVA program.

### Hypoxia induction

MEF WT and Akt DKO cells were plated in p6 culture dishes at a density of 1×10^5^ cells/ml. After approximately 18 h the culture dishes were placed in the hypoxic culture chamber (BioSpherix). The oxygen concentration in the chamber was maintained with an oxygen sensor (Proox Model 110) and a carbon dioxide sensor (ProCO_2_ model 120).

## Supporting Information

Figure S1 **Akt-IV induces the translocation of the ATF6 reporter in different cell lines.** (**A**) Diagram of pEYFP vector used for pYFP-NLS-mATF6short construction. ATF6 transmembrane (TmD) and luminal domains (LD) downstream of YFP were linked to the SV40 nuclear localization signal (NLS). The sites for ATF6 protease cleavage (S1P and S2P) are depicted. HeLa cells were transfected with YFP-NLS-mATF6short and GalT1-CFP plasmids, to label the Golgi apparatus. After 48 h, cells were treated with the ER stressor Tg for 4 h and then fixed and immunostained with antibodies against calnexin/Alexa Fluor® 594, to label ER. Yellow, YFP-NLS-mATF6short; Red, Calnexin (ER); Cyan, GalT1-CFP (Trans-Golgi); Blue, DNA; scale bar, 5 µm. The images showing ATF6 reporter in the nucleus (middle panel) or in the Golgi (bottom panel) are representative of the population response. (**B**) MCF7 (upper panel) and COS7 (lower panel) cells were transfected with the YFP-NLS-mATF6short plasmid. After 48 h, cells were treated with Akt-IV for 5 h and then fixed and imaged; scale bar, 5 µm. Data are representative of at least three independent experiments.
(TIF)

Figure S2 **Traditional inhibitors of the Akt pathway have marginal or no effect on PERK/eIF2α activation even at long times.** HEK293T cells were treated with DMSO (control), LY294002 (20 µM), Akt-VIII (5 µM) or Akt-IV (10 µM), for the indicated times. Protein extracts were analyzed by WB using the indicated antibodies. Data are representative of at least three independent experiments.
(TIF)

Figure S3 **Distribution of Akt isoform 3 in Akt DKO MEFs.** WT and Akt DKO MEF cells were fixed and immunostained with antibodies against (**A**) Akt/Alexa Fluor® 488 or (**B**) pAkt S473/Alexa Fluor® 488. Scale bar, 5 µm. Data are representative of at least three independent experiments.
(TIF)

Figure S4 **YFP-ATF6 and Akt-CFP colocalize in the ER.** (**A**) Diagram of pECFP-N1 vector used for pAkt1-CFP construction. (**B**) HeLa cells were transfected with YFP-NLS-mATF6short and Akt1-CFP plasmids. After 48 h, cells were fixed and imaged; scale bar, 5 µm. N: Nucleus; C: Cytosol; ER: Endoplasmic Reticulum; PM: Plasma Membrane. Data are representative of at least three independent experiments.
(TIF)

Figure S5 **pAkt substrate containing blebs induced by ATK-IV and Akt-VIII.** HeLa cells were treated for 5 min with Akt-IV (10 µM), Akt-VIII (5 µM) or were mock treated (C). Cells were fixed and immunostained for pAkt substrate/Alexa Fluor® 594; scale bar, 5 µm. The numbers show the percentage of cells displaying blebs in each condition. Data are representative of at least three independent experiments.
(TIF)
