# Supplementary figures and images for: Modulation of the Akt Pathway Reveals a Novel Link with PERK/eIF2α, which Is Relevant during Hypoxia
Source: PLoS One. 2013 Jul 29;8(7):e69668. doi: 10.1371/journal.pone.0069668 (PMC3726764; doi:10.1371/journal.pone.0069668)

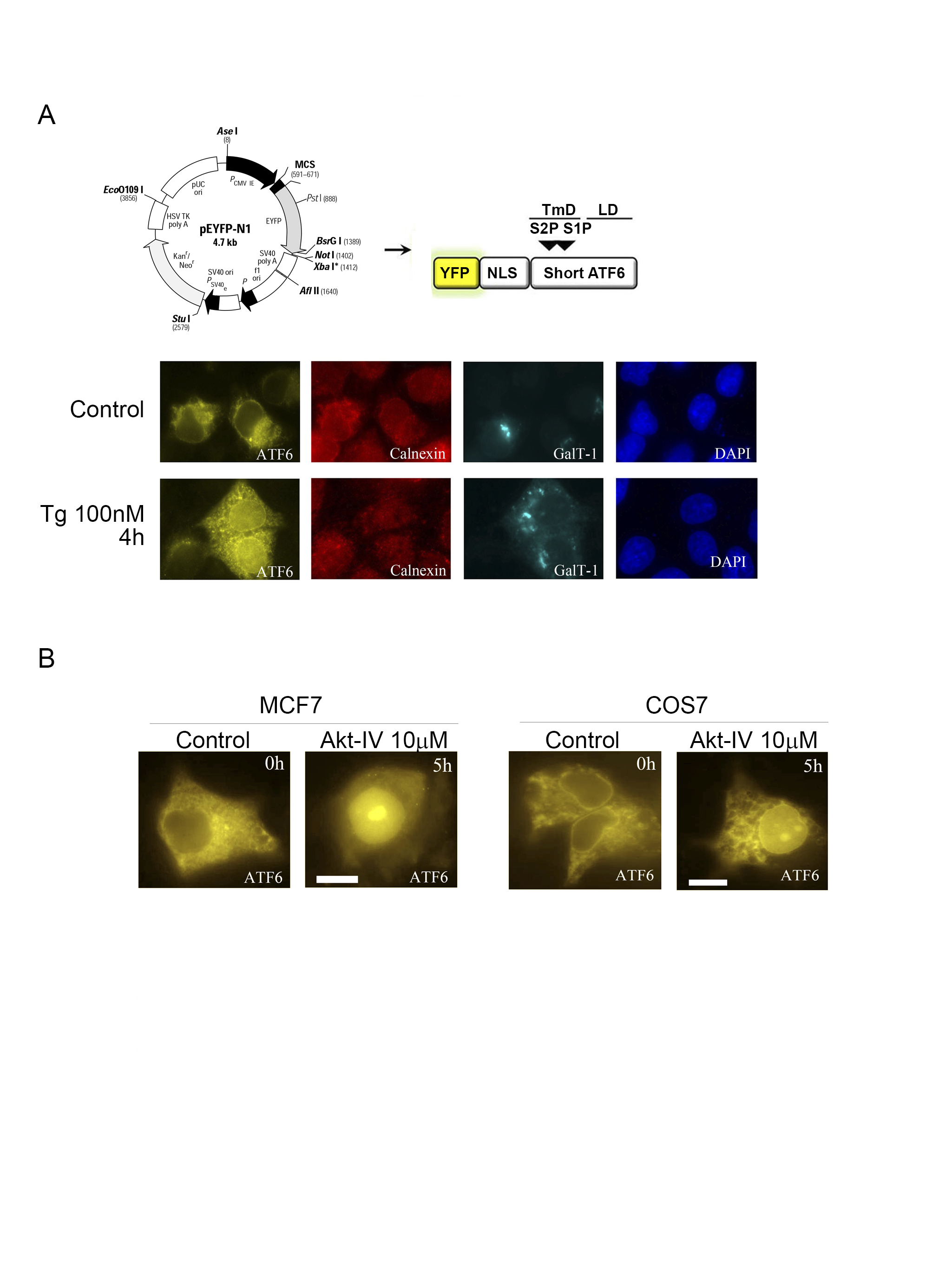

Supplement: Figure S1 — Akt-IV induces the translocation of the ATF6 reporter in different cell lines. (A) Diagram of pEYFP vector used for pYFP-NLS-mATF6short construction. ATF6 transmembrane (TmD) and luminal domains (LD) downstream of YFP were linked to the SV40 nuclear localization signal (NLS). The sites for ATF6 protease cleavage (S1P and S2P) are depicted. HeLa cells were transfected with YFP-NLS-mATF6short and GalT1-CFP plasmids, to label the Golgi apparatus. After 48 h, cells were treated with the ER stressor Tg for 4 h and then fixed and immunostained with antibodies against calnexin/Alexa Fluor® 594, to label ER. Yellow, YFP-NLS-mATF6short; Red, Calnexin (ER); Cyan, GalT1-CFP (Trans-Golgi); Blue, DNA; scale bar, 5 µm. The images showing ATF6 reporter in the nucleus (middle panel) or in the Golgi (bottom panel) are representative of the population response. (B) MCF7 (upper panel) and COS7 (lower panel) cells were transfected with the YFP-NLS-mATF6short plasmid. After 48 h, cells were treated with Akt-IV for 5 h and then fixed and imaged; scale bar, 5 µm. Data are representative of at least three independent experiments. (TIF) [file pone.0069668.s001.tif]

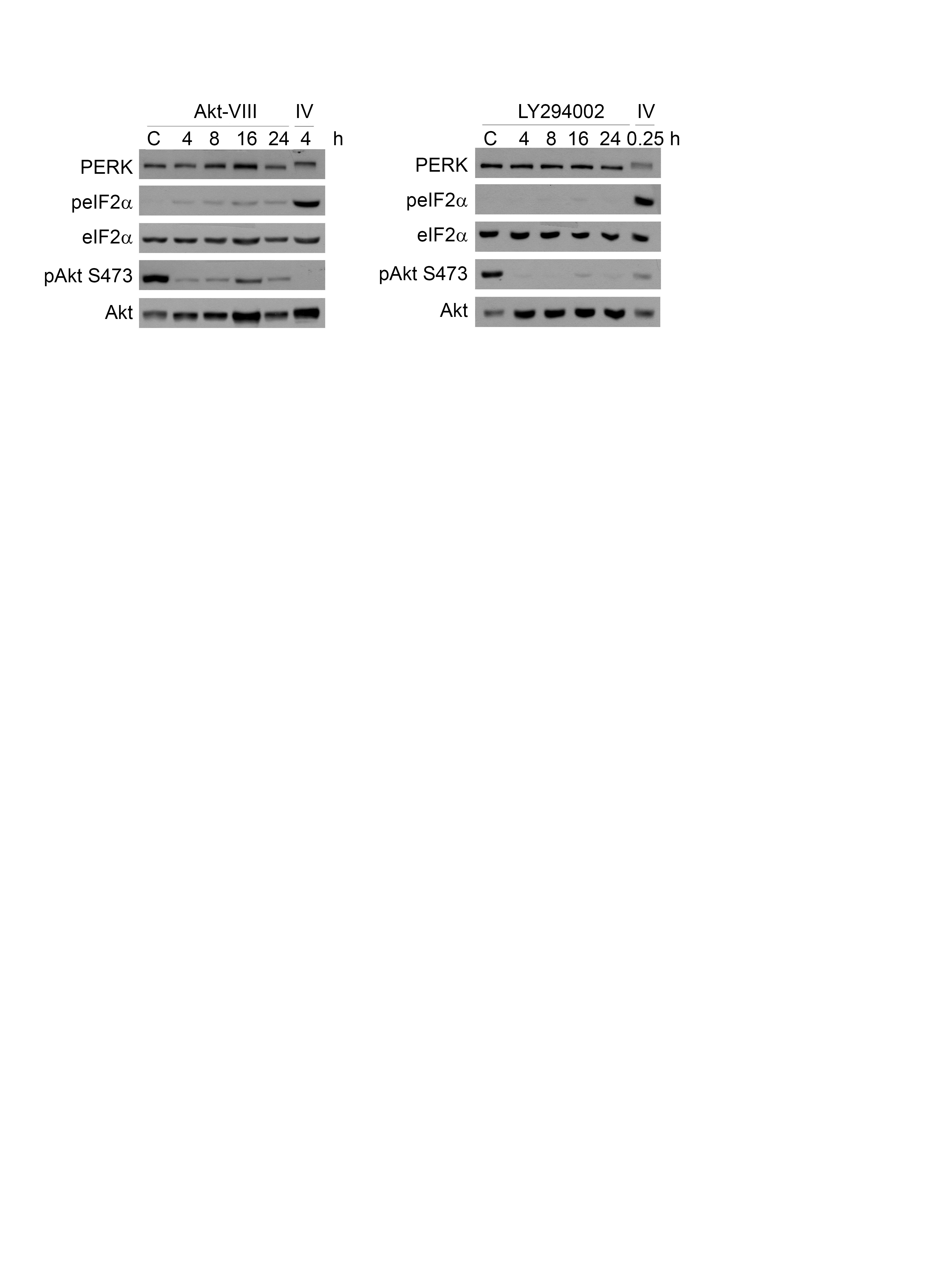

Supplement: Figure S2 — Traditional inhibitors of the Akt pathway have marginal or no effect on PERK/eIF2α activation even at long times. HEK293T cells were treated with DMSO (control), LY294002 (20 µM), Akt-VIII (5 µM) or Akt-IV (10 µM), for the indicated times. Protein extracts were analyzed by WB using the indicated antibodies. Data are representative of at least three independent experiments. (TIF) [file pone.0069668.s002.tif]

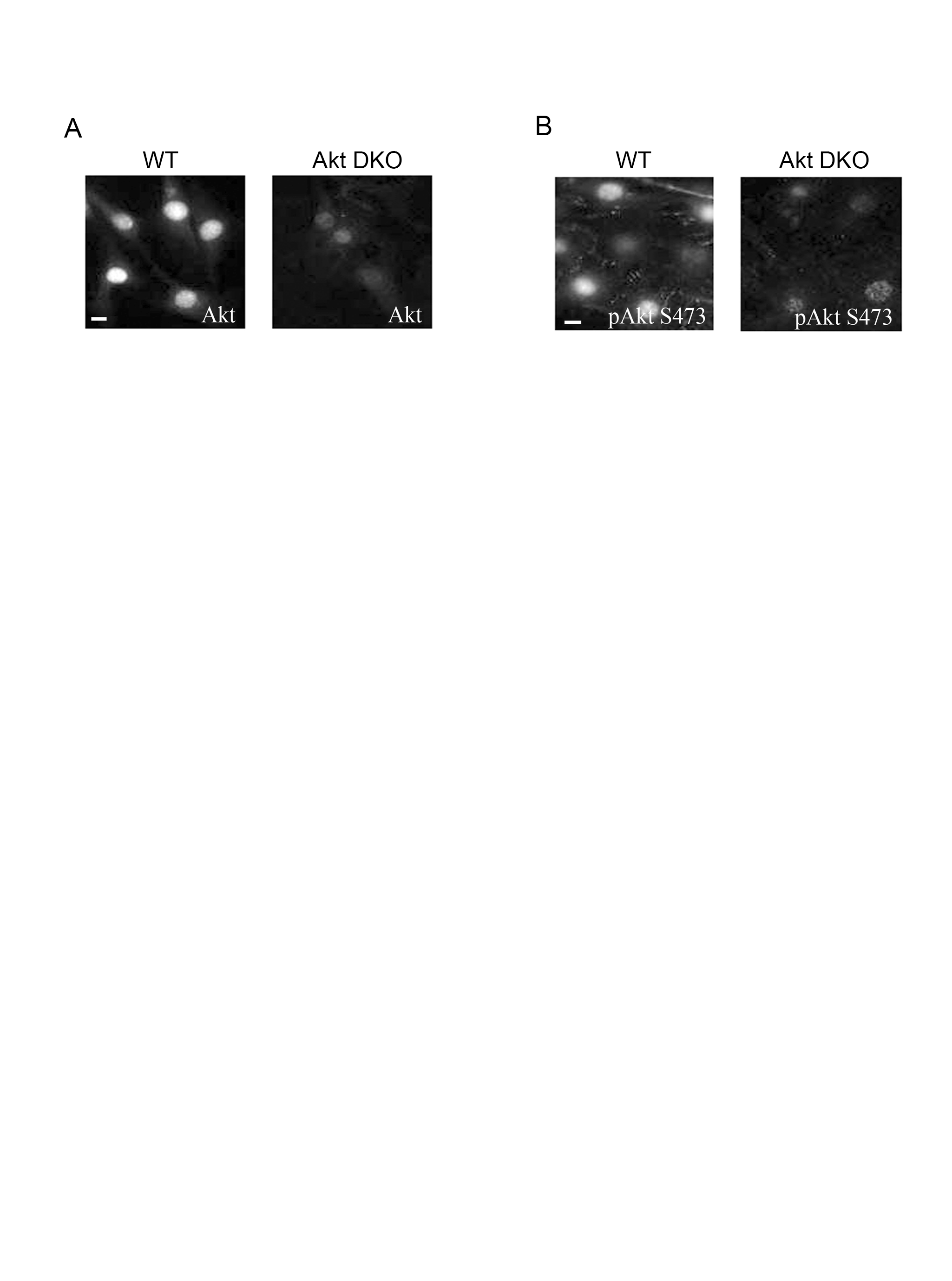

Supplement: Figure S3 — Distribution of Akt isoform 3 in Akt DKO MEFs. WT and Akt DKO MEF cells were fixed and immunostained with antibodies against (A) Akt/Alexa Fluor® 488 or (B) pAkt S473/Alexa Fluor® 488. Scale bar, 5 µm. Data are representative of at least three independent experiments. (TIF) [file pone.0069668.s003.tif]

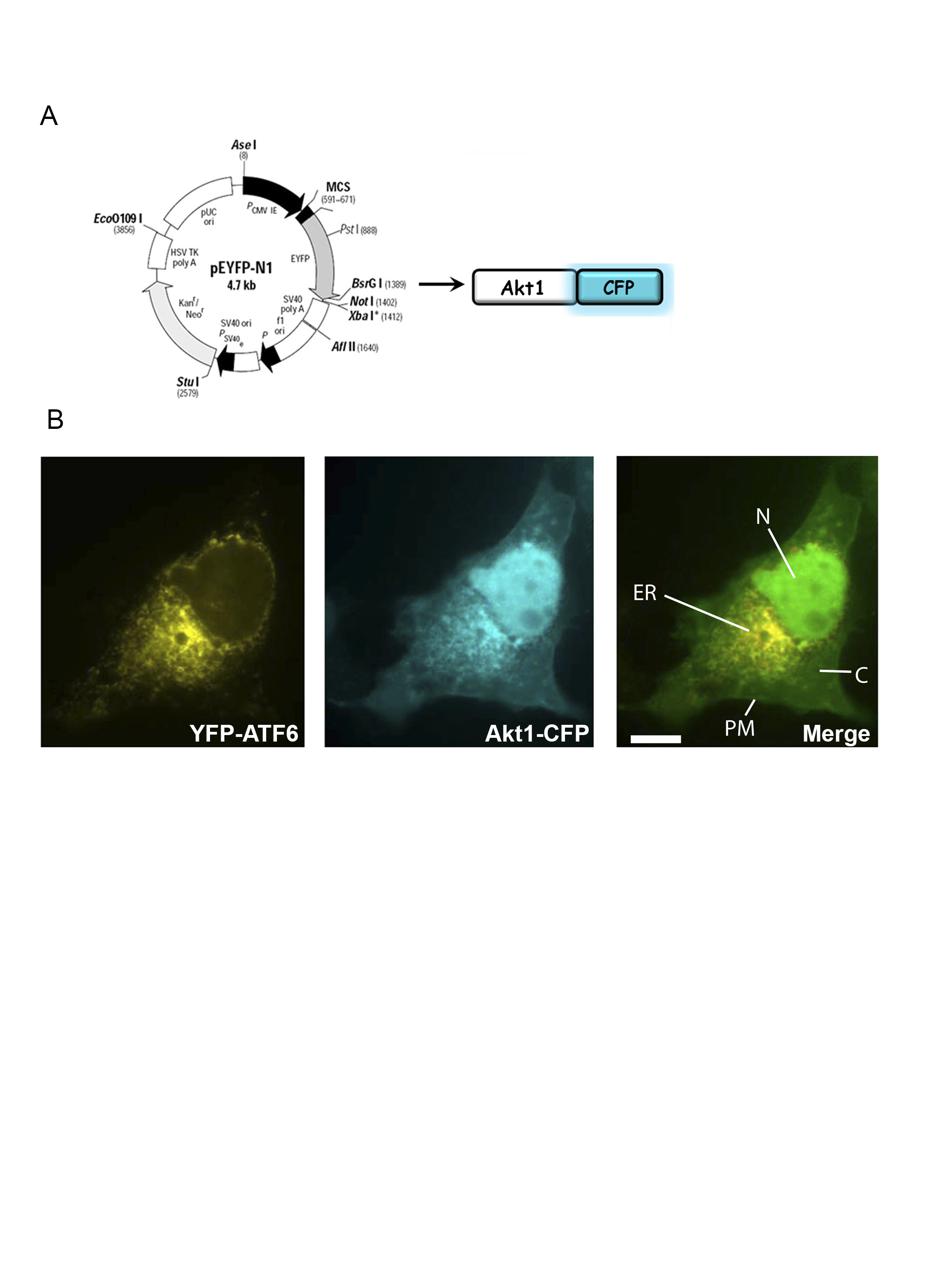

Supplement: Figure S4 — YFP-ATF6 and Akt-CFP colocalize in the ER. (A) Diagram of pECFP-N1 vector used for pAkt1-CFP construction. (B) HeLa cells were transfected with YFP-NLS-mATF6short and Akt1-CFP plasmids. After 48 h, cells were fixed and imaged; scale bar, 5 µm. N: Nucleus; C: Cytosol; ER: Endoplasmic Reticulum; PM: Plasma Membrane. Data are representative of at least three independent experiments. (TIF) [file pone.0069668.s004.tif]

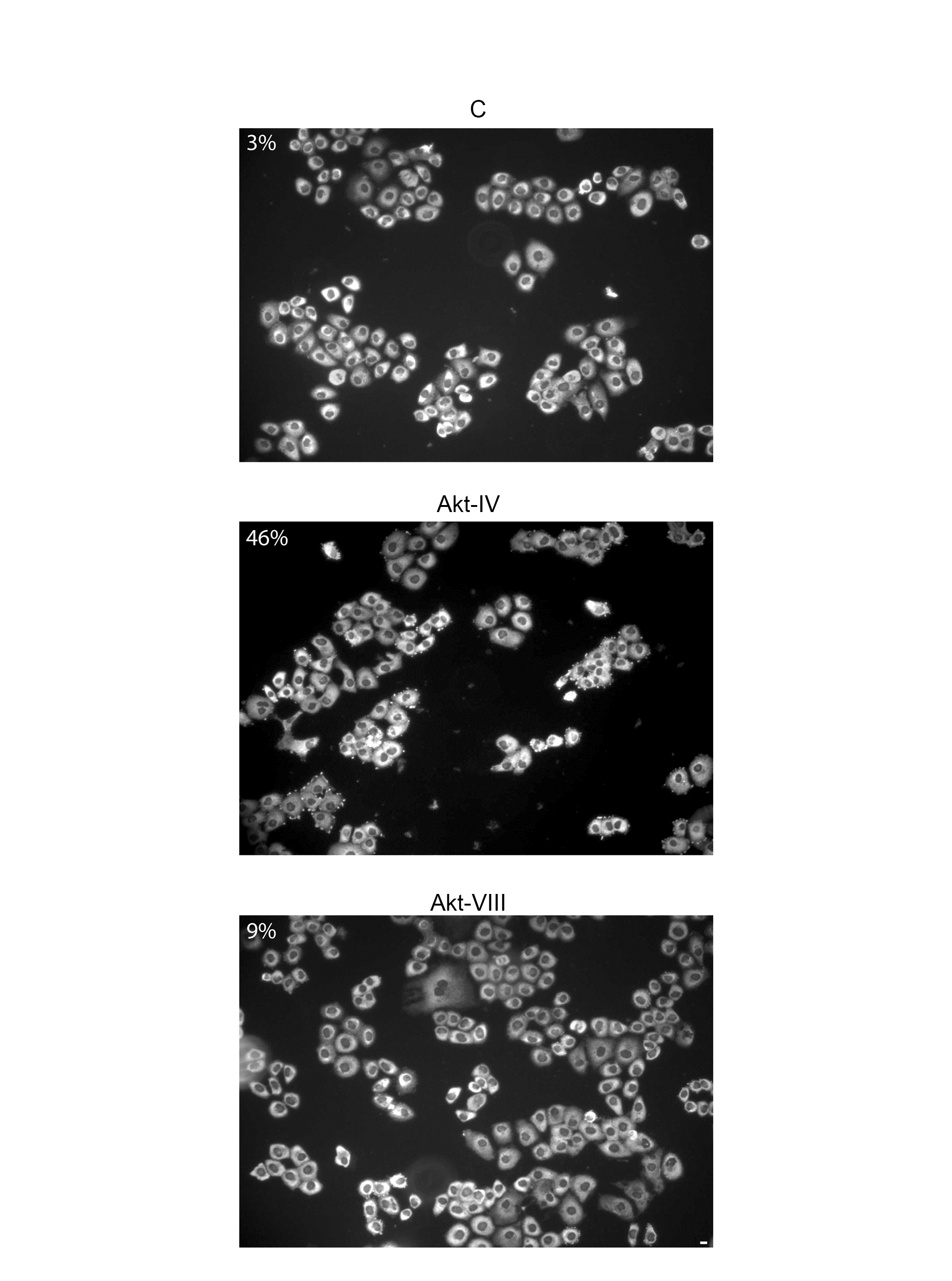

Supplement: Figure S5 — pAkt substrate containing blebs induced by ATK-IV and Akt-VIII. HeLa cells were treated for 5 min with Akt-IV (10 µM), Akt-VIII (5 µM) or were mock treated (C). Cells were fixed and immunostained for pAkt substrate/Alexa Fluor® 594; scale bar, 5 µm. The numbers show the percentage of cells displaying blebs in each condition. Data are representative of at least three independent experiments. (TIF) [file pone.0069668.s005.tif]
